# Morel-Lavallée Lesion of the Elbow Region ‎in a Young Male: Case Report and ‎Literature Review

**DOI:** 10.7759/cureus.27303

**Published:** 2022-07-26

**Authors:** Sarmad R Sulaiman, Abdullah M Alsuhaymi, Shadha ‎ A Al-Zubaidi, Alhanouf ‎ A Almusallam, Ahmed M Yassin, Rayan AlArabi

**Affiliations:** 1 Department of Orthopedic Oncology, Al Madinah Al Munawara Hospital, Madina, SAU; 2 Department of Orthopedic Surgery, King Fahad Hospital, Medina, SAU; 3 Department of Diagnostic Imaging, Madina Cardiac Centre, Madina, SAU; 4 Department of Orthopedic Surgery, King Salman Medical City, Al Madina Al Munawara Hospital, Madina, SAU; 5 Department of Orthopedic Surgery, King Salman Medical City,‎ Al Madina Al Munawara Hospital, Madina, SAU; 6 Department of Orthopedic Oncology, King Salman Medical City, Medina, SAU

**Keywords:** upper limb, post-traumatic ‎pseudocyst, post-traumatic extravasations, morel-lavallee lesion, degloving injury

## Abstract

The Morel-Lavallée lesion is a fluid collection resulting from the traumatic separation ‎of the ‎subcutaneous tissue from the underlying fascia. It frequently occurs over the trochanteric ‎region but ‎may also occur in the flank, lumbosacral region, and buttock. Morel-Lavallée lesions ‎in the upper limb are rarely reported in the literature.‎

In this report, we present a case of a 42-year-old male, not known to have ‎‎any medical ‎diseases, who suffered from a post-traumatic left elbow mass that had existed for seven months before his presentation to our clinic. It is worth reporting this case to increase the awareness of ‎this little-known pathology among orthopedic surgeons. In addition, most of the Morel-Lavallée ‎lesions mentioned in the literature are located in the lower limb.‎

## Introduction

Morel-Lavallée lesions (MLLs) are also referred to as post-traumatic soft tissue cysts, post-‎traumatic extravasations, Morel-Lavallée eﬀusions, or Morel-Lavallée seromas [1-3]. MLLs are closed degloving injuries combined with high-velocity trauma, crush ‎injuries, and blunt trauma, resulting in separation of the subcutaneous fat from the underlying fascia ‎leading to cavity formation associated with injury to the lymphatics and the blood capillaries in ‎the vicinity [1-3]. Eventually, the hematoma is ‎resorbed, and serosanguineous fluid appears [3]. Next, the serosanguineous collection resolves spontaneously or is subjected to an inflammatory reaction with a consequent ‎fibrous capsule formation filled with necrotic fatty tissue blood products and fibrin debris [1-3].‎ In general, MLLs present as gradually enlarged swelling associated with tautness, pain, and ‎cutaneous hypoesthesia or anesthesia because of the subdermal afferent nerve damage [1,3]. ‎Moreover, the fluctuance on palpation is an important clinical feature that helps in accurate ‎diagnosis and correlation with the history [1,3]. Unfortunately, no typical histopathologic findings of MLLs were reported in the literature [2]. ‎Therefore, the diagnosis is based on a physical examination and radiological investigations, ‎mainly magnetic resonance imaging (MRI), the investigation of choice for this lesion [2-4]. ‎A small number of MLLs in the upper limb are reported in the literature reviews ‎in PubMed and Google Scholar.

## Case presentation

A 42-year-old man was referred to our orthopedic oncology surgery clinic for a left ‎elbow mass. He sustained blunt trauma to his elbow after he slipt and fell down on the ground seven months before the visit. Subsequently, two days after ‎the trauma, he noted a feeling ‎of fullness in the elbow that had since persisted. The patient was later followed up at the fracture clinic and was diagnosed clinically with traumatic bursitis, which was managed with a compressive bandage. Later ‎he began noting a progressive enlargement of the ‎mass at the posteromedial aspect of the elbow over the next two weeks, which ‎interfered with his day-to-day activities ‎during elbow flexion or extension. The area was otherwise ‎asymptomatic. There was no medical ‎history of malignancy, and no fevers, chills, or night sweats.‎

**Figure 1:**
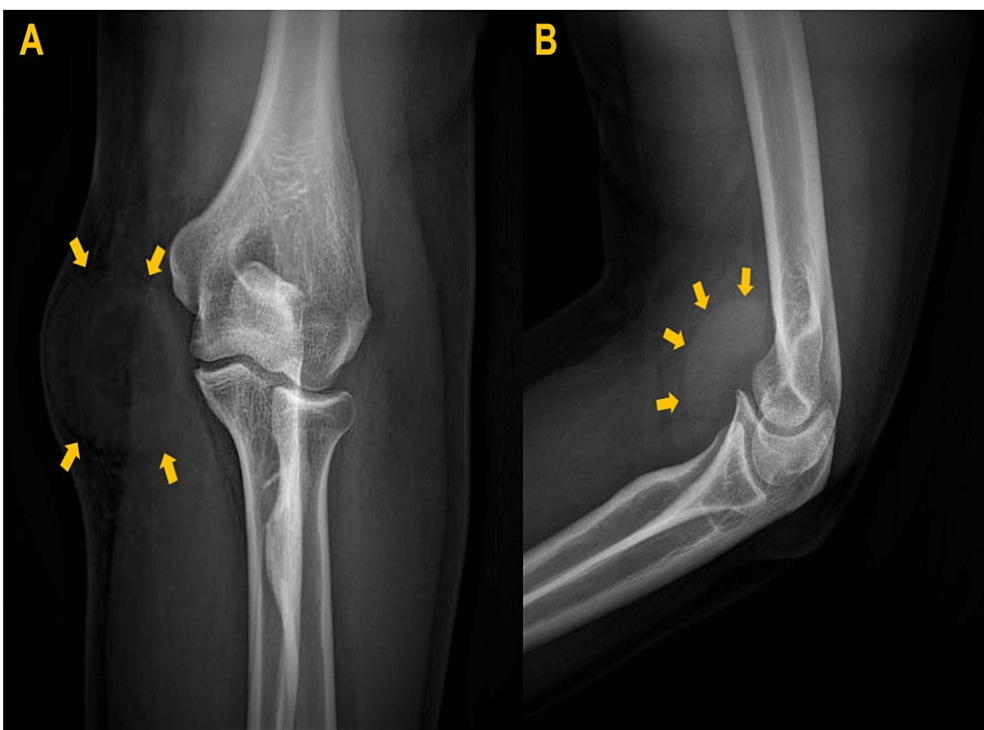
(A) An elbow radiograph (anterior-posterior view) shows a ring-like soft ‎tissue ‎mass at the ‎medial aspect of the elbow (yellow arrows). ‎(B). An elbow ‎radiograph (lateral view) shows a soft tissue mass (yellow arrows).‎

**Figure 2:**
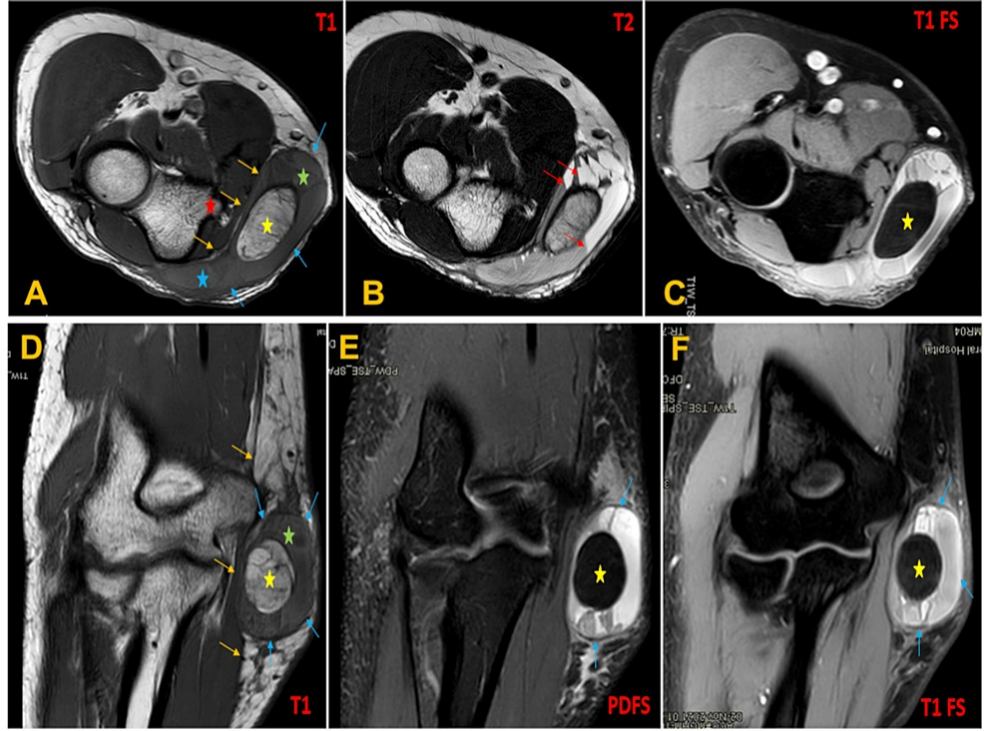
Selected multiplanar, multisequence MRI of the left elbow using ‎intravenous gadolinium.‎ (A, D) Axial T1-weighted and coronal T1-weighted images at the same level as Figure A show a left elbow mass that overlies the fascia (yellow arrows) in the medial epicondyle region (red star) with a tail-like expansion (blue star) extended posteriorly and fusing with the surrounding fascia. The mass ‎appeared with two zones: a high-intensity central oval shape zone, which represents a fatty ‎mass (yellow star), and a hematoma, which displays a homogeneous hyperintense signal to skeletal muscle ‎‎(green and blue stars), surrounded by low-intensity peripheral pseudocapsule (blue arrows).‎ (B) Axial T2-WI at the same level as Figure A reveals multiple fluid-fluid levels indicating the chronicity of the ‎lesion (red arrows) ‎with no evidence of internal enhancement.‎ (C) Axial T1-weighted fat-saturated image at the same level as Figure A ‎displays the suppression of the central fatty ‎mass (yellow star) with heterogeneous hyperintense signal outer to the central mass. (E, F) Coronal proton density (PD) fat saturation (FS) and coronal T1-weighted fat-saturated image sequences at the same level as Figure A show the signal suppressed central fatty ‎mass (yellow star) with heterogeneous ‎intermediate to hyperintense signal outer to it‎, surrounded ‎with low-intensity peripheral pseudocapsule (blue arrows).‎

On the clinical examination, he looked overall healthy in appearance. The mass was centered over the humerus’s medial condyle. ‎The overlying skin was normal. On palpation, the mass was non-tender and mobile, ‎with a soft consistency and with no areas of ‎induration. The elbow range of motion was full, and ‎the vascular, motor, and sensory examinations distally were normal. The ‎blood tests (complete blood count‎, biochemistry, C-reactive protein, and erythrocyte sedimentation rate) were unremarkable. Plain radiograph demonstrated a ring-like soft ‎tissue mass (Figure 1). We ‎did not feel that the US study was enough for the diagnosis; therefore, MRI was requested. The ‎MRI study with intravenous (IV) gadolinium of the left elbow showed a ‎‎well-defined, large, cystic lesion overlying the fascia (Figure 2). The lesion measures ‎approximately 45x75x25 millimeters (longitudinal x anterior-posterior x transverse).

The history, physical examination, and imaging findings were consistent with a left elbow region ‎‎MLL. As the lesion was disrupting the ‎patient’s activities of daily living, he ‎requested treatment.‎ ‎

**Figure 3:**
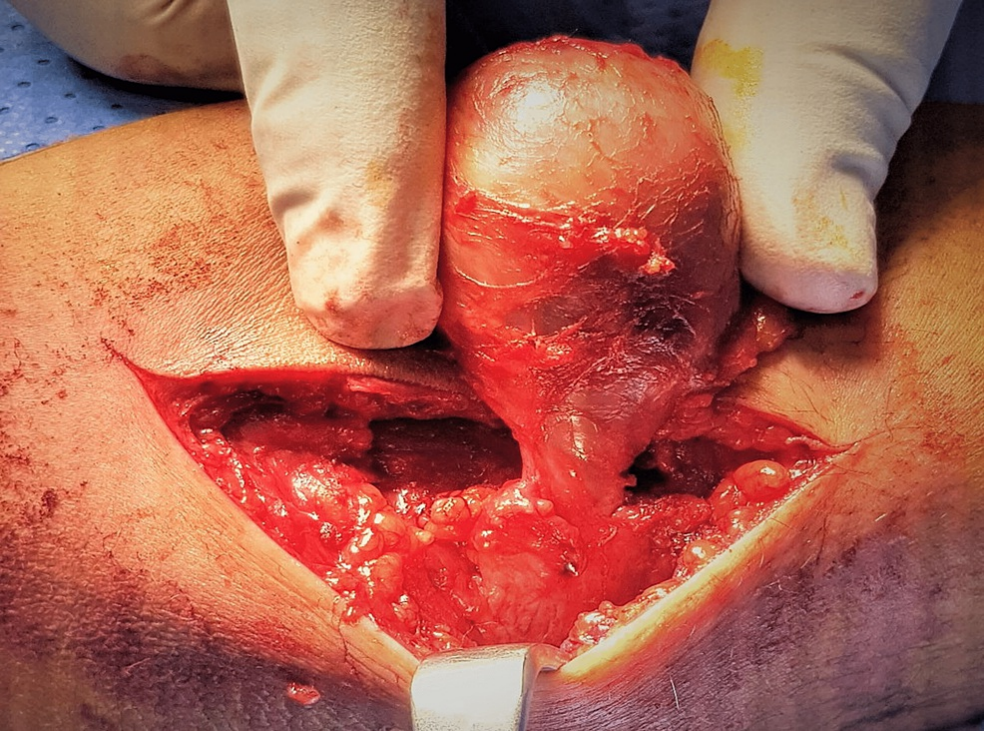
Photograph showing the surgical approach to removing the mass.‎

**Figure 4:**
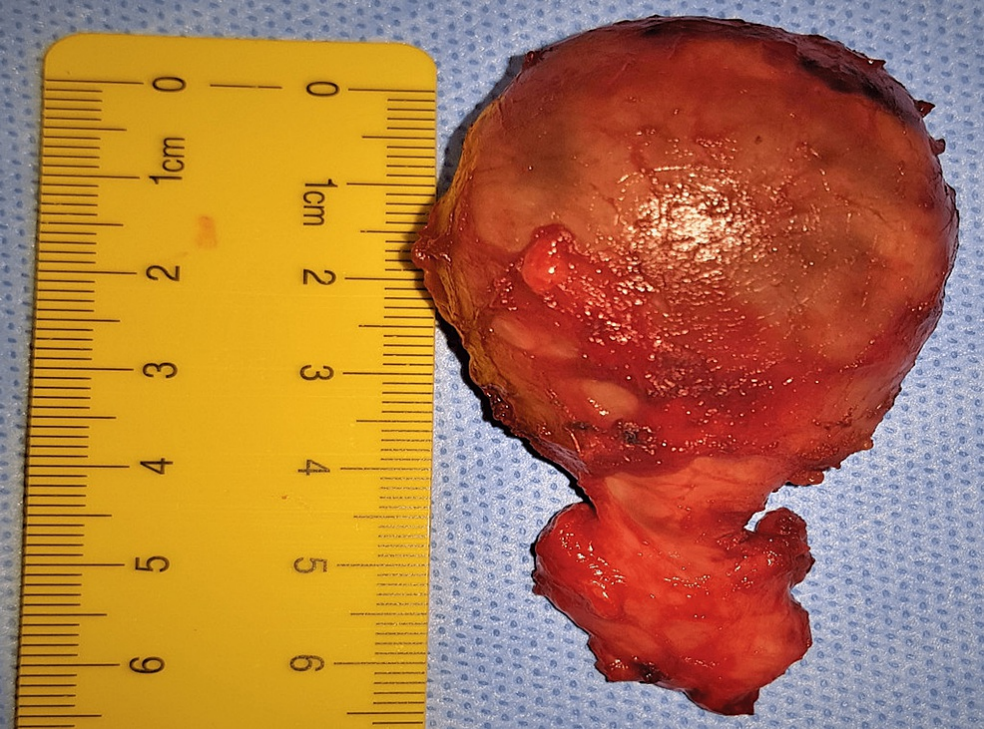
Gross pathology image showing the cystic lesion encapsulated and has a tail-like expansion.‎

**Figure 5:**
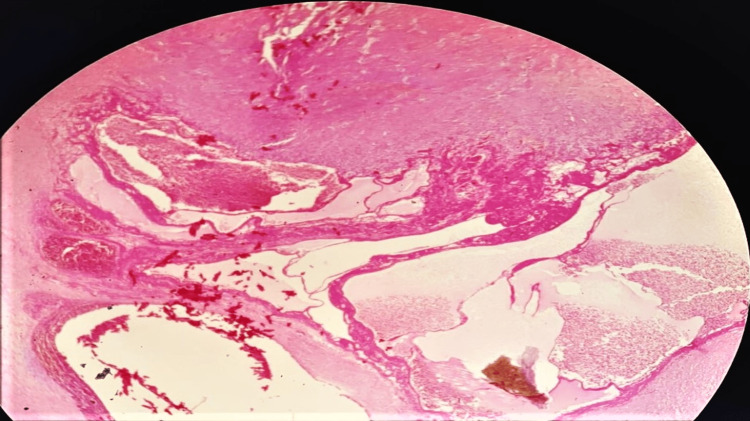
Histopathology photograph demonstrating an organized hematoma with ‎bleeding, dilated ‎vessels, ‎fibrin exudate, fibrosis hyalinization, focal endothelial ‎proliferation, and ‎neovascularization.‎

A pneumatic tourniquet was applied to the upper ‎arm with the patient under general anesthesia. The lesion was removed through a medial approach (Figure 3). The assessment of the ‎excised specimen revealed a cystic mass with tail-like expansion surrounded by a fibrous capsule (Figure 4)‎. The ‎‎histopathology result demonstrated an organized hematoma with bleeding, dilated vessels, fibrin ‎exudate, fibrosis hyalinization, focal endothelial proliferation, and neovascularization (Figure 5). ‎The findings were consistent with MLL.‎ The postoperative course went smooth without wound complications or recurrence of the collection in the follow-up period, which extended up to eight months.

## Discussion

Most reported anatomical locations for MLLs are the thigh and the knee regions ‎‎‎[1,3,5,6]. The ‎commonest location is in the knee ( 32%), the greater trochanter (‎‎29%), and the anterolateral ‎compartment of the leg (23%) ‎‎‎[3,6]. Furthermore, less frequently reported sites include the scalp, scapular ‎region, arm, hand, abdominal wall, lumbosacral region, and the calf ‎‎‎[1,3,5,6]. For the MLLs, MRI ‎has been considered the modality of ‎choice due to its multiplanar acquirement, high contrast ‎resolution, the ability to determine hematoma chronicity, and the demonstration of detailed ‎anatomical features [2-4,6,7]. However, MRI assessment yields a variable degree of appearance that depends on age and blood product breakdown [2-4]. Currently, there is no traditional classification system for MLLs [8].‎ Several investigators classified MLLs according to the time of injury or the presence or absence of the capsule [8,9]. Additionally, MLLs were classified into six types depending on the MRI appearance [2-4,6,7]. Nevertheless, these classifications have limitations in guiding the treatment [1,2,6,8-10]‎‎‎. ‎Type I lesions are most frequently not encapsulated, representing a seroma with fluid-like signal intensity ‎‎(SI) [3,4,7,11].‎ Type II lesion resembles a subacute hemorrhage and displays a homogeneous hyperintense SI on both T1-weighted image (WI) and T2-WI [3,4,7,11]. Moreover, a hemosiderin-rich hypointense capsule often appears on T1-WI and T2-WI [3,4,7]. Occasionally, an internal ‎homogeneity is detected and attributed to fluid-fluid levels, fat globules entrapment, ‎or internal septations [3,4,7]. Type III lesions indicate chronicity and hematoma organization, demonstrating hypo- or intermediate SI on T1-WI and heterogeneous intermediate to hyperintense SI on T2-WI [3,4,7]. Finally, the next three types are long-standing lesions, which may display more atypical MRI features [3,4].‎ In type IV MLLs, a closed laceration to the fatty tissue is associated with a peri-fascial separation, with or ‎without a seroma or hematoma. [4,7]. Those lesions are not encapsulated [6,7] and exhibit a low SI on T1-WI and ‎high SI on T2-WI [3,4,7]. Type V MLLs are usually adjacent to the fascia and exhibit a tiny, round pseudo-nodular appearance. ‎Infrequently, they demonstrate irregular enhancement peripherally with skin retraction [3,4,7].‎ Type VI is considered when an infection occurs, leading to internal septations, capsular thickening, and edema in ‎the fascia and the nearby fatty tissue [3,4,7].‎‎ Unfortunately, ‎the diagnosis of MLL is often missed or delayed because MLL is relatively infrequently ‎encountered [2,8]‎.‎ Therefore, MLLs represent a diagnostic challenge, especially in an atypical ‎‎location [2-4,7,8].‎ Fortunately, the clinical history, physical examination, and MRI are essential to ‎differentiating most confounding pathologies [2,4,8]. The common differential diagnoses‎ are hematoma, abscess, and fat necrosis [2,6,8]. The imaging overlap between MLLs ‎and hematoma is understandable, but the lesion’s chronicity and location should ‎lead to the diagnosis of MLLs [2]. Moreover, an abscess usually exhibits peripheral ‎‎enhancement and contains air locules, which are not features of MLLs [2]. Of note, fat necrosis is ‎a small component of MLLs; however, MRI should be easily distinguished between solitary ‎fat necrosis and MLLs ‎[2,6,7,12,13]. Additionally, soft tissue sarcoma should be excluded in slow-growing soft tissue masses, especially with solid enhancing components ‎[2,4,6-8,14]. ‎Consequently, a biopsy should be performed in case of questionable or aggressive MRI features ‎[2].

Currently, there are no treatment guidelines for MLLs ‎[1,2].‎ Compressive ‎dressings are applied to prevent fluid aggregation and seal off the ‎dead space in conjunction with percutaneous fluid aspiration ‎[2,10]. ‎‎Compressive bandaging has been recommended in non-‎capsulated acute, small lesions ‎[1,2,4,10,15]. ‎On the other hand, percutaneous aspiration ‎for lesions with a volume of more than 50 ml is vulnerable to recurrence; thus, ‎multiple aspirations are usually required ‎[1,15]‎. Moreover, sclerotherapy is ‎recommended after failure of the percutaneous aspiration and has a success rate of ‎‎‎95.7% ‎[1,10,15]‎. Sclerotherapy effectively induces fibrosis, leading to obliteration of the ‎pathological cavity in the lesions with volumes up to 700 ml ‎‎‎[1,10,16]. In persistent and long-standing lesions, surgical debridement with pseudocapsule resection is a ‎suitable ‎option‎ [2,4,6]‎. Moreover, an absolute indication of surgery is in lesions associated ‎with an open fracture, skin necrosis, and deep infection [2,4,8,10]‎‎.

## Conclusions

MLL represents a serious‎ and infrequent soft tissue injury, ‎which is often delayed or misdiagnosed. Therefore, MRI is ‎the modality of choice for the diagnosis. It is essential to differentiate the lesion from ‎the other pathologies, especially soft tissue sarcoma in the slowly growing masses. ‎Consequently, radiologists should be aware of the different radiological findings ‎of the lesion. Furthermore, orthopedic surgeons should have suspicion when ‎managing patients after blunt or shearing injuries. Treatment is different; ‎compressive bandage is preferred for acute lesions, while percutaneous ‎aspiration, ‎sclerotherapy, and debridement are preferred for chronic lesions.‎

## References

[1] Singh R, Rymer B, Youssef B, Lim J (2018). The Morel-Lavallée lesion and its management: a review of the literature. J Orthop.

[2] McLean K, Popovic S (2017). Morel-Lavallée lesion: AIRP best cases in radiologic-pathologic correlation. Radiographics.

[3] De Coninck T, Vanhoenacker F, Verstraete K (2017). Imaging features of Morel-Lavallée lesions. J Belg Soc Radiol.

[4] Diviti S, Gupta N, Hooda K, Sharma K, Lo L (2017). Morel-Lavallee lesions-review of pathophysiology, clinical findings, imaging findings and management. J Clin Diagn Res.

[5] Cochran GK, Hanna KH (2017). Morel-Lavallee lesion in the upper extremity. Hand (N Y).

[6] Bonilla-Yoon I, Masih S, Patel DB (2014). The Morel-Lavallée lesion: pathophysiology, clinical presentation, imaging features, and treatment options. Emerg Radiol.

[7] Mellado JM, Bencardino JT (2005). Morel-Lavallée lesion: review with emphasis on MR imaging. Magn Reson Imaging Clin N Am.

[8] Greenhill D, Haydel C, Rehman S (2016). Management of the Morel-Lavallée lesion. Orthop Clin North Am.

[9] Carlson DA, Simmons J, Sando W, Weber T, Clements B (2007). Morel-lavalée lesions treated with debridement and meticulous dead space closure: surgical technique. J Orthop Trauma.

[10] Shen C, Peng JP, Chen XD (2013). Efficacy of treatment in peri-pelvic Morel-Lavallee lesion: a systematic review of the literature. Arch Orthop Trauma Surg.

[11] Mellado JM, Pérez del Palomar L, Díaz L, Ramos A, Saurí A (2004). Long-standing Morel-Lavallée lesions of the trochanteric region and proximal thigh: MRI features in five patients. AJR Am J Roentgenol.

[12] Tsai TS, Evans HA, Donnelly LF, Bisset GS 3rd, Emery KH (1997). Fat necrosis after trauma: a benign cause of palpable lumps in children. AJR Am J Roentgenol.

[13] Chan LP, Gee R, Keogh C, Munk PL (2003). Imaging features of fat necrosis. AJR Am J Roentgenol.

[14] Kumar S, Hasan R, Kadavigere R, Maddukuri SB, Puppala R (2015). Morel-Lavallee Lesion (MLL) mimicking a soft tissue neoplasm. J Clin Diagn Res.

[15] Nickerson TP, Zielinski MD, Jenkins DH, Schiller HJ (2014). The Mayo Clinic experience with Morel-Lavallée lesions: establishment of a practice management guideline. J Trauma Acute Care Surg.

[16] Bansal A, Bhatia N, Singh A, Singh AK (2013). Doxycycline sclerodesis as a treatment option for persistent Morel-Lavallée lesions. Injury.

